# EGFR inhibition by (-)-epigallocatechin-3-gallate and IIF treatments reduces breast cancer cell invasion

**DOI:** 10.1042/BSR20170168

**Published:** 2017-05-17

**Authors:** Fulvia Farabegoli, Marzia Govoni, Enzo Spisni, Alessio Papi

**Affiliations:** 1 Department of Pharmacology and Biotechnology (FaBiT), University of Bologna, Bologna, Italy; 2 Department of Specialistic, Diagnostic and Experimental Medicine (DIMES), University of Bologna, Bologna, Italy; 3 Department of Biological, Geological, and Environmental Sciences, (BiGea), University of Bologna, Bologna, Italy

**Keywords:** breast carcinoma, CD44, epidermal growth factor receptor, extracellular matrix metalloproteinase inducer, (-)-epigallocatechin-3-gallate (EGCG), 6-OH-11-O-hydroxyphenantrene (IIF)

## Abstract

Epidermal growth factor receptor (EGFR) expression is an important marker in breast carcinoma pathology and is considered a pivotal molecule for cancer cell proliferation, invasion and metastasis. We investigated the effects of epigallocatechin-3-gallate (EGCG), the most active green tea catechin, in combination with 6-OH-11-O-hydroxyphenanthrene (IIF), a synthetic retinoid X receptor-γ (RXRγ) agonist, on three breast carcinoma cell lines: MCF-7, MCF-7TAM and MDA-MB-231. EGFR and AKT activation and molecular markers of cell motility and migration (CD44, extracellular matrix metalloproteinase (MMP) inducer (EMMPRIN), MMP-2, MMP-9 and tissue inhibitor of metalloproteinases (TIMPs)) were studied after EGCG and IIF treatments. The EGCG + IIF treatment was the most active in down-regulating EGFR phosphorylation at Tyr^1068^ in all the investigated cell lines; p473AKT was also down-regulated in MCF-TAM cells. EGCG + IIF was also the most active treatment in reducing the expression of markers of invasion and migration in all the three cell lines: CD44, EMMPRIN, MMP-2 and -9 expression decreased, whereas TIMPs were up-regulated. Zymography and scratch assay also confirmed the reduced invasion tendency. We considered that EGCG and IIF treatments could alter the molecular network based on EGFR, CD44 and EMMPRIN expression interdependence and reduced the migration tendency in MCF-7, MCF-7TAM and MDA-MB-231 cells. These events only occurred in association with AKT inactivation in MCF-7TAM cells. In conclusion, the combination of EGCG and IIF significantly attenuated the invasive behaviour of breast carcinoma cells.

## Introduction

Green tea, obtained from the leaves of *Camellia sinensis*, is a popular beverage traditionally consumed in the Asia-Pacific region, particularly in China and Japan. Numerous epidemiological *in vivo* and *in vitro* studies suggest that the molecules present in green tea, chiefly catechins and the most abundant catechin epigallocatechin-3-gallate (EGCG), have many biological activities, including antioxidant, free radical scavenging and iron chelating properties [1–3]. Green tea catechin activity has also been widely associated with cancer prevention and treatment: epidemiological and laboratory studies have found tea consumption inversely associated with the onset and development of certain cancer types [4–7]. EGCG was found to behave as a multitarget molecule, with minimal or no side-effects, numerous sites of action and different mechanisms. Laboratory studies suggested that EGCG works mainly at cellular level by down-regulating molecules involved in cell proliferation (p21, p27, p53, Cyclin D and E, CDK2, 4 and 6) and apoptosis (Bax, Bcl-2 and p53). EGCG also targets molecules that regulate cell motility, invasion and metastasis, such as the HER family, vascular endothelial growth factor receptor (VEGFR), matrix metalloproteinases (MMPs), 67-kDa laminin receptor (67LR) and the phosphatidylinositol-3-kinase (PI3K)-Akt pathway [8–12]. Furthermore, EGCG is an interesting molecule for adjuvant combined therapies, as it can improve the efficacy of chemotherapeutics currently used in cancer therapy (tamoxifen, doxorubicin and cisplatinum) by promoting synergistic cytotoxic effects [13–16]. The strategy of combining EGCG with chemotherapeutics might become a potential vehicle to reduce drug-related toxicities in patients treated for cancer. Two of our previous studies showed that when EGCG was given in association with a synthetic retinoid X receptor-γ (RXRγ) agonist named 6-OH-11-O-hydroxyphenanthrene (IIF) to breast carcinoma [17], cholangiocarcinoma and colorectal carcinoma cell lines, cytotoxicity increased [18]. RXR-selective compounds have been found to have antitumour effects in mammary carcinoma, NSCLCs, myeloid leukemic cells, and prostate cancer cells without the dose-limiting toxicities associated with other retinoids. These compounds are currently under investigation as therapeutic agents in the treatment of cancer, particularly in tamoxifen-resistant breast cancer [19,20]. EGCG and IIF combined treatments elicited apoptosis by inhibiting the AKT survival pathway and activating pro-apoptotic Foxo3a [17]. The PI3K-Akt pathway is a key cascade downstream of membrane-bound receptor tyrosine kinases, including the epidermal growth factor receptor (EGFR) family and can be regulated by phosphorylation, which activates and represses a multitude of downstream molecules [21]. EGFR/AKT activation has been demonstrated to control molecules associated with cell proliferation, epithelial–mesenchymal transition (EMT), cell motility and invasion [22].

The present study better defined the molecular pathways modulated by the combined treatments of EGCG and IIF in three breast carcinoma cell lines: MCF-7, MCF-7TAM and MDA-MB-231. We investigated the effects of EGCG and IIF treatments on EGFR and AKT activation and several markers of cell motility and migration: CD44, extracellular MMP inducer (EMMPRIN), MMPs and tissue inhibitor of metalloproteinases (TIMPs), which are directly regulated by EGFR and AKT and mark the tendency of neoplastic cells to invade and metastasize [23,24].

## Materials and methods

### Cell lines

MCF-7 and MDA-MB-231 were purchased from the American Type Culture Collection (Rockville, MD, U.S.A.) and grown in E-MEM (MCF-7) or D-MEM (MDA-MB-231) supplemented with 10% FBS, 2 mM L-glutamine, 50 U/ml penicillin, 50 μg/ml streptomycin and grown at 37°C in a humidified atmosphere with 5% CO_2_. MCF-7 cells resistant to tamoxifen (MCF-7TAM) were selected by growing MCF-7 cells in MEM medium without Phenol Red, containing 2 mM L-glutamine, 50 U/ml penicillin, 50 μg/ml streptomycin, 10 % FBS serum charcoal treated and 10^−7^ M 4-OH-hydroxytamoxifen as previously described^ 17^[17]. In order to avoid any risk of contamination, the cell lines were assayed for oestrogen receptor α (ERα) expression by reverse transcription-PCR (RT-PCR) and immunoflurescence every 3 months: MCF-7 expressed ERα to a great extent, whereas MDA-MB-231 was completely devoid of ERα expression. The MCF-7TAM cell line was grown in 10^−7^ M 4-OH-hydroxytamoxifen, which is cytotoxic for both MCF-7 and MDA-MB-231 cells. Cell lines were routinely tested for mycoplasma infection by fluorescence microscope inspection after DAPI staining.

### Reagents

EGCG, 4-Hydroxytamoxifen, L-glutamine, penicillin-streptomycin, sulphorhodamine B, MEM medium without L-glutamine and Phenol Red, DAPI and 1,4-diazabicyclo[2.2.2]octane (DABCO), were all purchased from Sigma–Aldrich, MO, U.S.A. IIF (pat. WIPO W0 00/17143), was provided by Dr K. Ammar, Bologna, Italy. E-MEM, D-MEM and FBS were purchased from the Lonza group, Basel, Switzerland. Formalin 40% was from Carlo Erba, Milano, Italy. Antibodies: anti-EGFR, anti-AKT and anti-p473AKT, were all from Thermo Scientific, Waltham, MA, U.S.A., anti-p1068EGFR from Novex, Life Technologies, Carlsbad, CA, U.S.A., anti-mouse-FITC conjugated and anti-γ-tubulin (Sigma–Aldrich, MO, U.S.A.), anti-p308AKT (Rockland Immunochemicals, Pottstown, PA, U.S.A.), anti-CD44 (BD Bioscience, San Jose, CA, U.S.A.), anti-EMMPRIN (Zymed Lab, San Francisco, CA, U.S.A.), anti-MMP-2, MMP-9 and anti-TIMPs (all from Santa Cruz, Dallas, TX, U.S.A.), anti-rabbit and anti-mouse-peroxidase conjugated antibodies (GE Healthcare, Milano, Italy).

### EGCG storage and treatments

EGCG was dissolved in water with 3% ascorbic acid and stored at –20°C in aliquots. According to Hong and co-workers [25], EGCG is soluble in water and it is more stable in mild acidic conditions than at neutral pH. Ascorbic acid minimizes the EGCG autoxidation that may occur after dilution in complete medium before treatments.

### Western blot analysis

The cells were treated with EGCG and/or IIF for 24 h. After treatments, the cells were scraped and centrifuged at 300×***g*** for 10 min. The pellets were suspended in lysis buffer (20 mM Tris/HCl, pH 7.5, 0.5 mM EDTA, 0.5% Triton X-100, 5 mM Na_3_VO_4_) and sonicated on ice in the presence of protease inhibitors. Protein concentration was determined by the method of Lowry. Cell lysates (50 μg of protein per lane) were size-fractioned in 10% SDS-polyacrylamide before transfer to Hybond TM-C extra membranes (GE Healthcare, Milano, Italy) by standard protocols. The membranes were blocked with 5% milk in transfer buffer saline (TBS) at RT for 2 h and later they were incubated with the antibodies overnight at 4°C. The following antibodies, dissolved in TBS-5% milk were used: anti-EGFR, anti-p^1068^Tyr EGFR, anti-AKT, anti-p473AKT and anti-p308AKT, anti-CD44, EMMPRIN, MMP-2, MMP-9 and TIMPs. The membranes were washed twice with TBS-5% milk and incubated for 1 h with the respective peroxidase–conjugated antibodies. The primary antibodies were diluted 1:500 and the anti-rabbit or anti-mouse peroxidase–conjugated antibodies were diluted 1:1000. The proteins were detected by luminol. Bands were quantified by using densitometric image analysis software (Imagemaster VDS, Pharmacia Biotechnology, Piscataway, NJ, U.S.A.). Protein loading was controlled by anti-actin (1:1000) or anti-γ-tubulin (1:1000) detection. Experiments were performed in triplicate, normalized against actin or γ-tubulin control and statistically evaluated.

### Immunofluorescence

Cells were grown on coverslips. After treatments, the cells undergoing EGFR immunostaining were fixed in cold absolute methanol for 15 min, air-dried and then washed twice in PBS. The cells undergoing CD44 immunostaining were fixed in 1% formalin in PBS for 20 min and then in 70% ethanol, air-dried and washed twice in PBS. The samples were incubated in 10% BSA (Sigma–Aldrich, MO, U.S.A.) in PBS for 30 min at 37°C and subsequently in the primary antibody: mouse monoclonal antibody anti-EGFR (1:800 in 1% BSA in PBS) or mouse monoclonal antibody anti-CD44 (1:600 in 1% BSA in PBS) overnight at 4°C. Negative controls were also included: the primary antibody was eliminated from the solution incubated overnight at 4°C to assay the selective antigen localization. After washing, the samples were incubated in 1:800 (1% BSA in PBS) anti-mouse-FITC–conjugated secondary antibody for 1 h at 37°C, washed, air-dried and mounted in a solution of 1:500 DAPI in DABCO and analysed by a Nikon fluorescent microscope equipped with filters for FITC, TRITC and DAPI.

### RNA extraction and reverse transcription-PCR

Total RNA was extracted from harvested cells by guanidinium-phenol-chloroform method as described by Chomczynski and Sacchi [26] and quantified spectrophotometrically.

EGFR gene expression was evaluated by RT-PCR (Verso 1-Step RT-PCR Kit, Thermo Fisher Scientific, Monza, Italy) using β-actin as an internal reaction control.

**EGFR**; **F**: 5′-CTCACGCAGTTGGGCACTTT-3′, **R**: 5′-TCATGGGCAGCTCCTTCAGT-3′; **β-actin; F:** 5′-ATCGTGCGTGACATTAAGGAGAAG-3′, **R:** 5′-AGGAAGGAAGGCTGGAAGAGTG-3′. Annealing temperature was 58°C. PCR products were loaded on to a 1% agarose gel, run into an electrophoresis chamber, stained by Ethidium Bromide and visualized with a UV-transilluminator. Bands were analysed by Kodak Electrophoresis Detection and Analysis System (EDAS 290, Eastman Kodak Company, Rochester, NY, U.S.A.).

### Zymography

Cells were seeded and after 18 h, were placed in serum-free medium with EGCG and/or IIF for 24 h. MMP-2 and MMP-9 activity was determined by gelatin zymography as previously described [27]. MMP activities, indicated by clear bands of gelatin digestion on a blue background, were quantified by using densitometric image analysis software (Quantity One, Bio–Rad, Milano, Italy).

### Monolayer scratch assay

MCF-7, MCF-7TAM and MDA-MB-231 cells were grown until 80% confluence in 3.5-cm dishes and scratched using a P1000 pipette tip to create a cross at the centre of the plate. Then, the cells were treated as already described. Photos of the scratches were taken at 0, 24, 48 and 72 h to monitor scratch closure. The distance migrated was calculated on the basis of the reduction in the scratch width with respect to the zero hour time point using ImageJ software.

### Statistical analysis

All experiments were performed in triplicate. Statistical significance was assessed by ANOVA multiple comparison test with S.D., as appropriate, using PRISM 5.1 (GraphPad Software). The levels for accepted statistical significance were *P*<0.05 and *P*<0.01.

## Results

### EGCG and IIF treatments down-regulated EGFR expression and phosphorylation at Tyr^1068^

We demonstrated that EGCG treatment was cytotoxic to breast cell lines *in vitro* [17,27]. In two previous studies, on the basis of dose-response curves, we found that combinations of suboptimal EGCG and IIF concentrations significantly increased cytotoxicity: additive or synergistic effects were detected in breast [17], colorectal and colangiocarcinoma cell lines [18]. We found that 25 μg/ml EGCG and 15 or 30 μM IIF concentrations were significantly more cytotoxic than individual treatments in breast carcinoma cells. Apoptosis also increased significantly in the cotreated breast carcinoma samples with respect to individual treatments [17]. The present study investigated whether the combination of 25 μg/ml EGCG and 15 or 30 μM IIF could be effective in down-regulating EGFR and markers of migration and invasion in three breast carcinoma cell lines differing in biomolecular characteristics.

**Figure 1:**
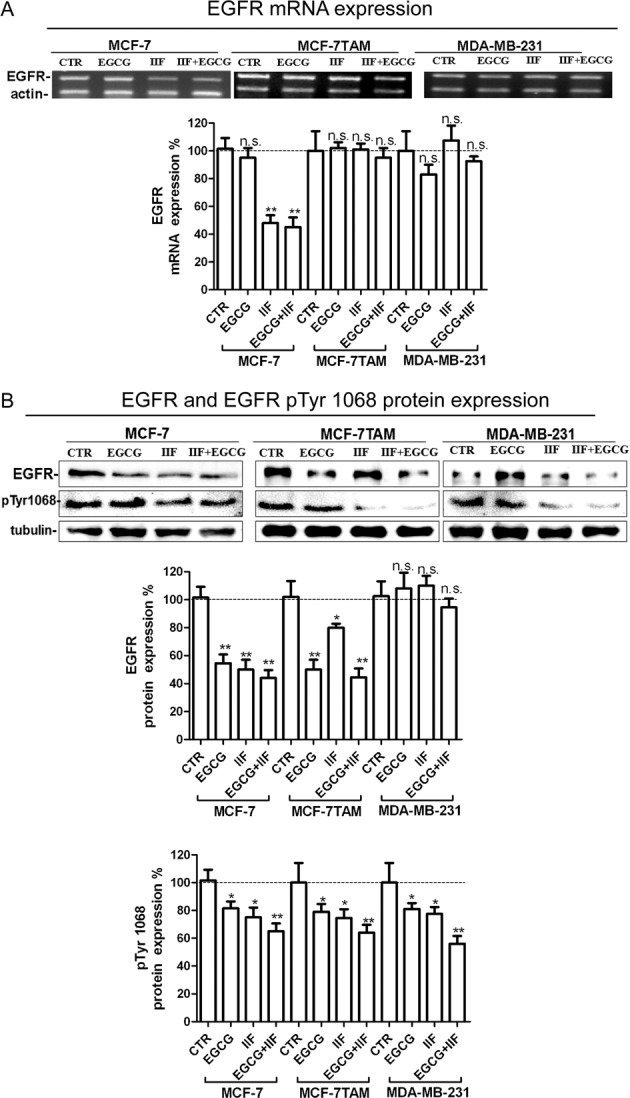
EGFR receptor expression in MCF-7, MCF-7TAM and MDA-MB-231 cells (**A**) RT-PCR for EGFR and β-actin (internal standard). RNA was isolated after 24 h treatments at the following concentrations: EGCG 25 μg/mL, IIF 15 μM in MCF-7 cells or 30 μM in MCF-7TAM and MDA-MB-231 cells and combined treatments EGCG + IIF at the aforementioned concentrations. *EGFR* mRNA expression was expressed as percentage of treated on control samples (CTR). Each bar represents the mean (± S.D.) of three independent experiments. n.s., not significant. *P<0.05; **P<0.01. (**B**) Western blot for EGFR and p1068EGFR: proteins were isolated after 24-h treatments at the following concentrations: EGCG 25 μg/mL, IIF 15 μM in MCF-7 cells or 30 μM in MCF-7TAM and MDA-MB-231 cells and combined treatments of EGCG + IIF at the aforementioned concentrations. EGFR protein expression was expressed as percentage of treated on control samples (CTR). γ-tubulin was used as an internal standard. Each bar represents the mean (± S.D.) of three independent experiments. n.s., not significant. *P<0.05; **P<0.01.

EGFR expression was investigated by RT-PCR, Western blot and immunofluorescence. Firstly, we observed that *EGFR* mRNA expression decreased sharply after IIF and IIF + EGCG treatments in the MCF-7 cell line, whereas it did not change in MCF-7TAM cells or MDA-MB-231 (Figure 1A). Indeed, Western blot revealed that EGFR protein dramatically decreased in MCF-7 and MCF-7TAM cells after all the treatments (Figure 1B). In addition to the 170-kDa band, corresponding to the EGFR full-length form, MCF-7 cells also expressed EGFR low MW bands (results not shown), possibly corresponding to a low molecular weight EGFR variant, named EGFR mLEEK [28]. EGFR immunostaining was attenuated on the cytoplasmic membrane in MCF-7TAM and MDA-MB-231 cells after EGCG treatment, whereas MCF-7 cells showed prevalent cytoplasmic localization (Supplementary Figure S1). Omission of primary antibody did not result in any detectable immunostaining (Supplementary Figure S2). EGFR activity was investigated using a specific antibody for Tyr^1068^ (pTyr^1068^EGFR) located at the C-terminal tail that is important for signal transduction. Tyr^1068^ is one of the major sites of EGFR autophosphorylation and is considered a marker of EGFR activity [29]. We found that pTyr^1068^ EGFR was down-regulated in all the investigated cell lines after all the treatments, and the combined treatment was the most effective (Figure 1B). Hence, the EGCG + IIF treatments were able to down-regulate EGFR activity independent of the different EGFR abundance and regulation in the three different cell lines.

### EGCG and IIF treatments down-regulated AKT and AKT phosphorylation at 473 serine (p^473^Ser) and 308 threonine (p^308^Thr)

**Figure 2:**
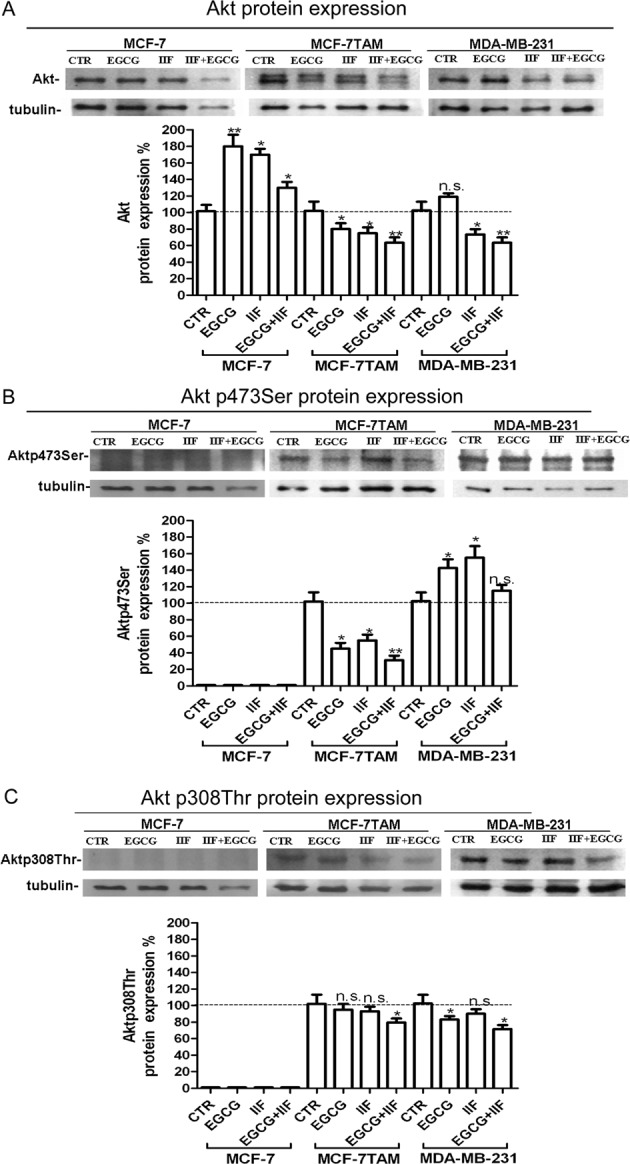
AKT, p473 AKT and p308 AKT expression in MCF-7, MCF-7TAM and MDA-MB-231 cells (**A**) Western blot for AKT: proteins were isolated after 24-h treatments at the following concentrations: EGCG 25 μg/mL, IIF 15 μM in MCF-7 cells or 30 μM in MCF-7TAM and MDA-MB-231 cells and combined treatments of EGCG + IIF at the aforementioned concentrations. AKT protein expression was expressed as percentage of treated over control samples (CTR). Each bar represents the mean (± S.D.) of three independent experiments. n.s. not significant. *P<0.05; **P<0.01. (**B**,**C**) Western blot for p473AKT and p308 AKT: proteins were isolated after 24-h treatments at the following concentrations: EGCG 25 μg/mL, IIF μM in MCF-7 cells or 30 μM in MCF-7TAM and MDA-MB-231 cells and combined treatments EGCG + IIF at the aforementioned concentrations. p473AKT and p308AKT protein expression were expressed as percentage of treated over control samples (CTR). Each bar represents the mean (± S.D.) of three independent experiments. n.s., not significant. *P<0.05 ; **P<0.01.

EGFR can regulate AKT, one of the most important signalling pathways activated in human cancer. AKT activation occurs by phosphorylation, chiefly at Ser^473^ and Thr^308^, which triggers cascades of further downstream phosphorylation events that modulate several aspects of cancer cell behaviour. AKT protein was expressed in all the three cell lines investigated, as detected by Western blot (Figure 2). The effects of EGCG and IIF given individually and in combination differed in the three cell lines. AKT expression increased in MCF-7 after EGCG and IIF individual treatments and was reduced by all treatments in MCF-7TAM and after IIF and EGCG + IIF in MDA-MB-231 cells (Figure 2A). p^473^SerAKT was down-regulated in MCF-7TAM after all the treatments, whereas p^308^ThrAKT expression decreased after EGCG + IIF treatment (Figure 2B). Interestingly, MCF-7 showed faint poorly detectable bands corresponding to p^473^SerAKT and p^308^ThrAKT (Figure 2C). MDA-MB-231 showed decreased expression in p^308^ThrAKT after EGCG and EGCG + IIF treatments. In conclusion, MCF-7TAM only showed a significant EGFR and AKT inactivation after EGCG and IIF treatments.

### EGCG and IIF attenuated the expression of molecular markers of invasion and impaired MCF-7, MCF-7TAM and MDA-MB-231 cell migration *in vitro*

We investigated the expression of CD44, EMPRINN, MMP-2, MMP-9 and TIMPs after EGCG and IIF treatments and performed a migration scratch test to investigate whether EGCG and IIF treatments could limit the acquisition of an invasive phenotype and behaviour in MCF-7, MCF-7TAM and MDA-MB-231 cells. These molecules were considered suitable markers of invasion and are correlated with EGFR activation [30,31].

**Figure 3:**
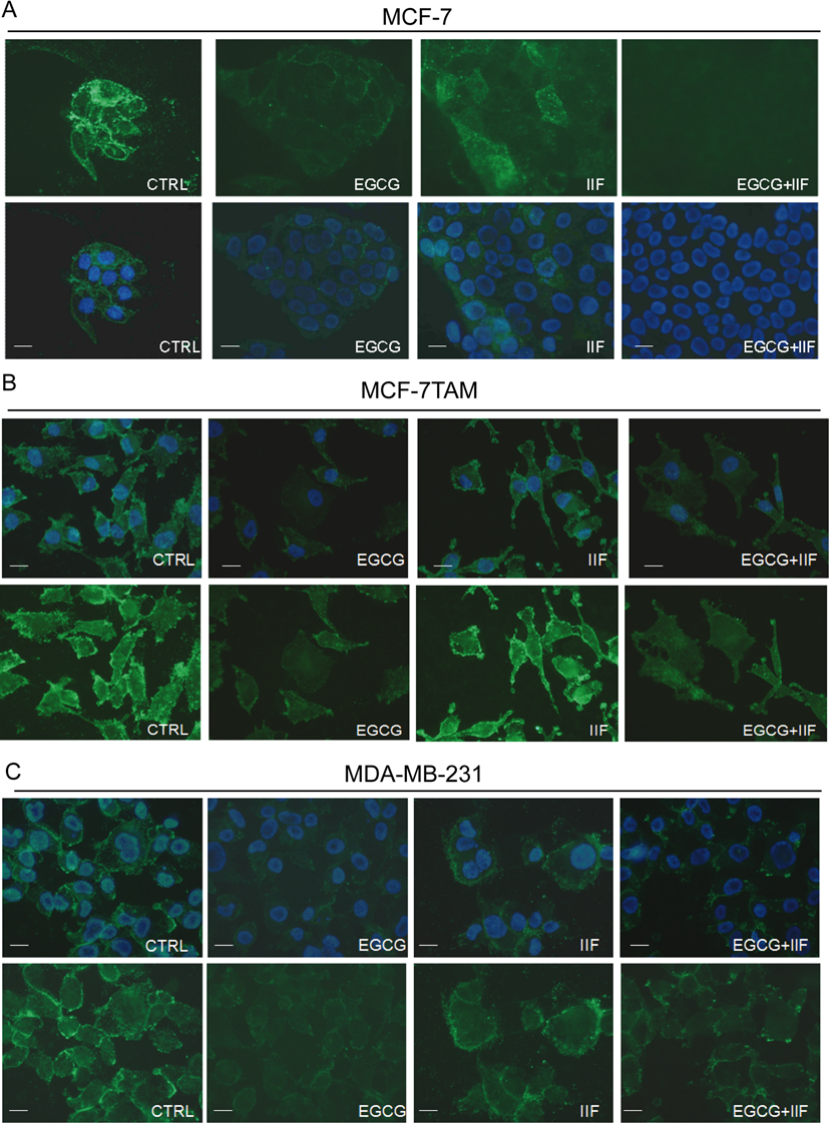
Immunofluorescence detection of CD44 in MCF-7, MCF-7TAM and MDA-MB-231 cells Control and 24 h treated cells were fixed in 1% formalin in PBS and in 70% ethanol. The samples were incubated with the primary anti-CD44 antibody overnight at 4°C. After washing, the cells were incubated with a secondary FITC–conjugated antibody. Nuclear DAPI staining in blue. Original magnification ×60.

**Figure 4:**
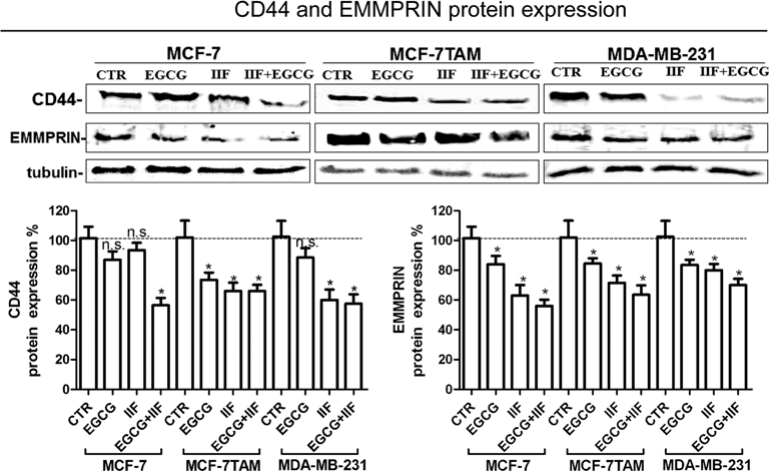
CD44 and EMMPRIN expression in MCF-7, MCF-7TAM and MDA-MB-231 cells Western blot for CD44 and EMMPRIN: proteins were isolated after 24-h treatments at the following concentrations: EGCG 25 μg/mL, IIF 15 μM in MCF-7 cells or 30 μM in MCF-7TAM and MDA-MB-231 cells and combined treatments of EGCG + IIF at the aforementioned concentrations. CD44 and EMMPRIN protein expression were expressed as percentage of treated over control samples (CTR). Each bar represents the mean (± S.D.) of three independent experiments. n.s., not significant. *P<0.05; **P<0.01.

First, we analysed CD44 expression in MCF-7, MCF-7TAM and MDA-MB-231 cells by immunofluorescence and Western blot. CD44 is a glycosylated protein highly expressed at the cell surface of breast, ovarian and prostate cancer cells including cancer stem cell (CSC) [32]. CD44 was clearly expressed at the surface of all the cell lines investigated (Figure 3). EGCG treatment (individually and in combination with IIF) efficiently suppressed CD44 immunostaining in all the cell lines investigated but MDA-MB-231 cells: we observed that after EGCG + IIF treatment, some residual MDA-MB-231 cells showed intense staining, among a cell population showing a weak fluorescence signal. IIF individual treatment weakened the immunostaining in MCF-7 cells (Figure 3). Western blot showed that the EGCG + IIF treatment was the most effective in down-regulating CD44 protein expression (Figure 4).

**Figure 5:**
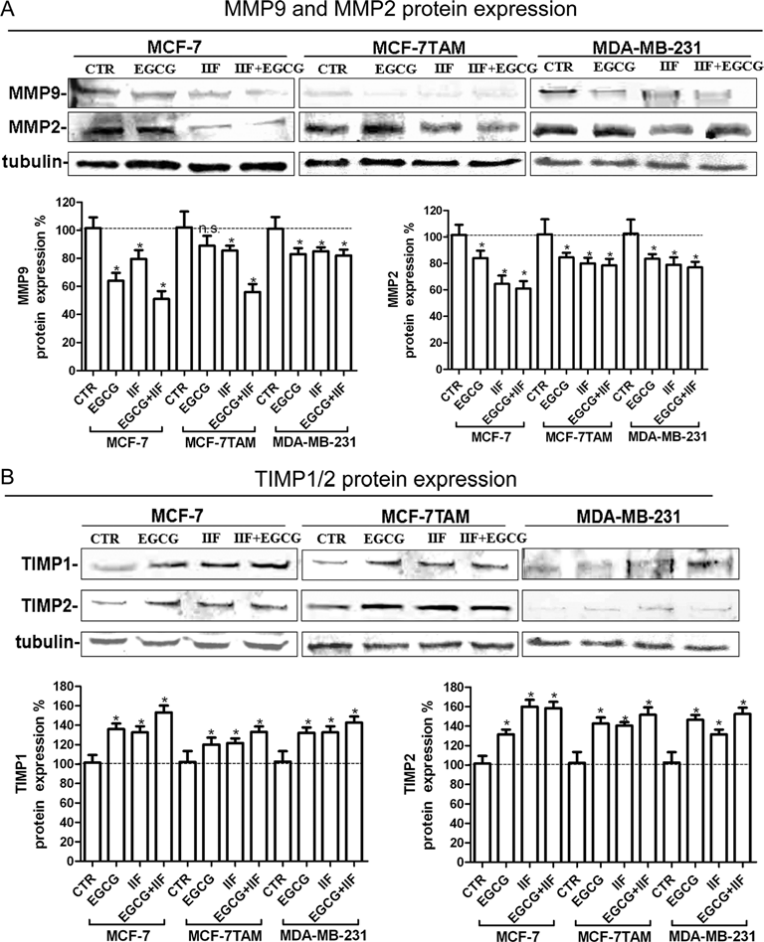
TIMP and MMP expression in MCF-7, MCF-7TAM and MDA-MB-231 cells (**A**,**B**) Western blot for TIMPs and MMPs: proteins were isolated after 24-h treatments at the following concentrations: EGCG 25 μg/mL, IIF 15μM in MCF-7 cells or 30 μM in MCF-7TAM and MDA-MB-231 cells and combined treatments of EGCG + IIF at the aforementioned concentrations. TIMP-1 and TIMP-2 (A) and MMP-2 and MMP-9 (B) protein expression were expressed as percentage of treated over control samples (CTR). Each bar represents the mean (± S.D.) of three independent experiments. n.s., not significant. *P<0.05; **P<0.01.

**Figure 6:**
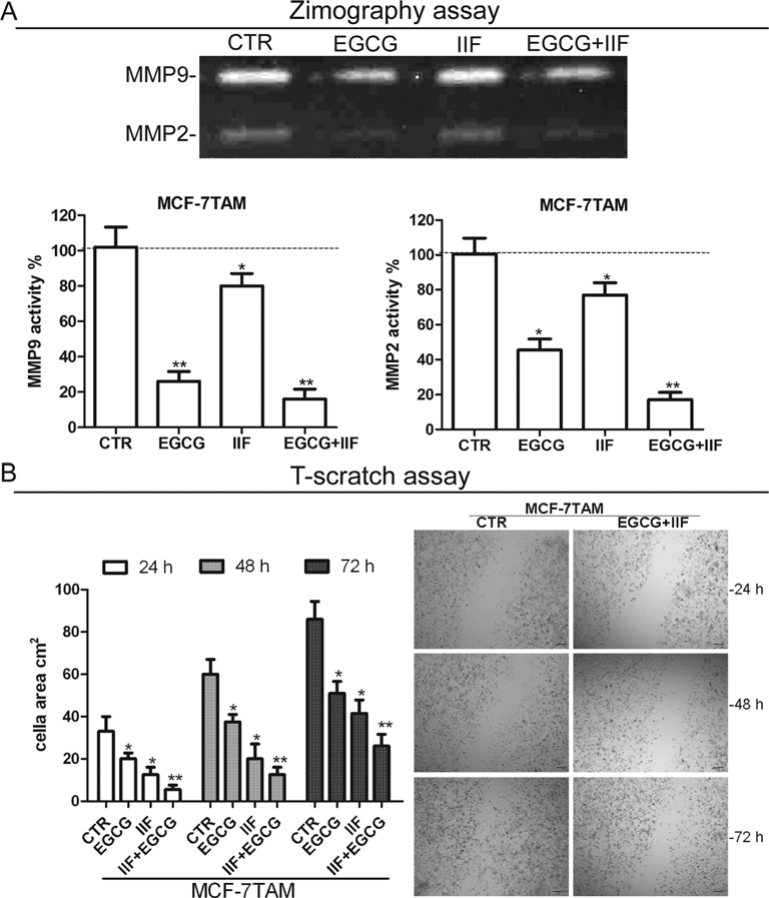
Zymography and scratch assay in MCF-7TAM cells (**A**) Zymography on MCF-7TAM cells: MMP-2 and MMP-9 activity was measured after 24-h treatments at the following concentrations: EGCG 25 μg/mL, IIF 15 μM in MCF-7 cells or 30 μM in MCF-7TAM and MDA-MB-231 cells and combined treatments of EGCG + IIF at the aforementioned concentrations. The gelatin zymogram showed MMP-2 (62 kDa) and MMP-9 (83 kDa) in serum-free conditioned media. CTR, control samples. (**B**) *In vitro* scratch assay on MCF-7TAM cells treated with EGCG 25 μg/mL + IIF 30 μM for 24 h and evaluated after 24, 48 and 72 h. Data are expressed as the number of cells per field. CTR, control samples. Each bar represents the mean (± S.D.) of three independent experiments. n.s., not significant. *P<0.05; **P<0.01. Original magnification ×20.

Hyaluronan–CD44 interaction up-regulates EMMPRIN, a molecule belonging to the Ig superfamily present on the plasma membrane of normal and cancer cells and induces breast epithelial cell invasiveness by promoting EGFR signalling and assembly with EGFR and CD44 in lipid raft like domains. We found that the inhibition of pTyr^1068^EGFR activity was correlated with down-regulation of EMMPRIN in all the cell lines investigated, after both individual and combined treatments (Figure 4). Furthermore, TIMPs, MMP-2 and MMP-9 expression was inversely correlated in all the three cell lines (Figure 5). MMPs are enzymes that able to degrade ECM proteins and process numerous bioactive molecules: they are known to play a critical role in the invasive behaviour of breast cancer cells. In particular, MMP-2 and MMP-9 were found to be up-regulated in metastatic cancer cell lines [33]. TIMP1 and TIMP2 are endogenous regulators of MMP-9 and MMP-2 respectively: in fact, we detected an increase in TIMP expression after the treatments. Zymography demonstrated a major reduction in MMP-2 and -9 proteins in the medium after EGCG + IIF treatments in all the three cell lines (Figure 6A, Supplementary Figure S3A), although the MMP-2 band was very weak in MDA-MB-231 cells. To study cell migration *in vitro*, we performed a scratch assay. Cell migration was impaired in all the cell lines investigated after all the treatments, but the most effective inhibitory effect was achieved after combined treatments in a time-dependent manner (Figure 6B, Supplementary Figure S3B).

## Discussion

Targeted therapies are currently the focus of tailored cancer treatment: the discovery of multiple molecular targets in cancer has increased the number of assayed molecules selectively directed to stop the malignant cell signalling pathways. These molecules are less toxic than conventional chemotherapeutics and they could target the malignant cell signal transduction machinery with minimal side-effects. The present study combined a widely investigated phytochemical present in green tea, EGCG, which has been demonstrated to have chemopreventive and therapeutic effects, and a synthetic derivative of all *trans*-retinoid acid (ATRA) named IIF, demonstrated to be a selective RXRγ inhibitor [34]. The EGCG + IIF combination was demonstrated to be cytotoxic to breast carcinoma [17], cholangiocarcinoma and colorectal carcinoma cells [18]. Since, we intended to investigate the molecular targets involved in the cytotoxic effects, in the present study, we did not change EGCG and IIF concentrations. The EGCG + IIF combination was more effective than individual treatments and we could treat cells with a low EGCG concentration, comparable with those measurable after consuming 1600 mg EGCG a day in capsules without side-effects [35]. Furthermore, recent improvements in the field of polymeric carrier systems with controlled release (nanoparticles, liposomes and micelles) enhanced the stability and bioavailability of EGCG and might improve EGCG oral absorption [36]. These new approaches might also overcome EGCG oxidation that occurs *in vitro*, although EGCG uptake in cell culture occurs very rapidly and make the molecule more stable than in the medium [37].

Apart from the expected selective effects on RXRγ receptors in the present study, EGCG and IIF were found to down-regulate pTyr^1068^EGFR. These findings are noteworthy as EGFR is one of the most frequently deregulated signalling pathways in human cancer and strictly correlated with invasion and metastasis. EGFR is an important marker in breast carcinoma pathology, a potential target of new therapies [38].

EGCG is thought to suppress EGFR activity by disturbing the “lipid raft” and therefore impairing EGFR dimerization and activation [39]. Tyr^1068^EGFR phosphorylation is considered critical for this function, since it has been implicated in receptor recycling after lipid raft alteration and regulation of CSC-related gene expression [40]. In the present study, Tyr^1068^ EGFR phosphorylation decreased in all the cell lines after all treatments, EGCG + IIF being the most effective of all.

EGCG and IIF treatments were also able to down-regulate molecular pathways downstream from EGFR, although with remarkable differences in the three cell lines. EGFR and AKT interplay may be considered potentially responsible for CD44, EMPRIMM and MMPs down-regulation in MCF-7TAM cells. In fact, MCF-7TAM only showed a significant EGFR and AKT inactivation after EGCG and IIF treatments. AKT promotes cancer cell invasion via increased motility and MMPs production [41]. Indeed, AKT down-regulation did not occur in MCF-7 and MDA-MB-231 cells after EGCG and IIF treatments. In our hands, MCF-7 weakly expressed 170-kDa EGFR, as reported by Li and co-workers [42], whereas EGFR low MW bands were more abundant and visible. Furthermore, both p^473^SerAKT and p^308^ThrAKT bands were faint and poorly detectable. In MDA-MB-231 cells, p^308^ThrAKT phosphorylation was only down-regulated after individual treatments, although p^1068^TyrEGFR, MMP-2, CD44 and EMPRIMM were all down-regulated. Therefore, we speculated that CD44, EMPRIMM and MMP-2 (and to a lesser extent, MMP-9) down-regulation were not dependent on AKT inactivation in MCF-7 and MDA-MB-231 cells, but they occurred as a consequence of lipid raft disorganization that impaired EGFR dimerization and activation after treatments [39]. Tyr^1068^EGFR phosphorylation is considered critical for this function since it has been implicated in receptor recycling after lipid raft alteration [40]. EGFR can activate cancer cell motility and invasion by regulating CD44 and EMMPRIN [31]. CD44 is a receptor for hyaluronic acid (HA), collagens and MMPs: adhesion with HA plays an important role in cell migration and invadopodia formation [32]. EMMPRIN also induces breast epithelial cell invasiveness by promoting EGFR signalling and assembly with EGFR and multiprotein complexes including CD44 and MMPs in lipid raft domains [43]. In turn, EMMPRIN and CD44 can phosphorylate Tyr^1068^ EGFR, establishing a regulatory loop that fosters cancer cell proliferation and invasion [31]. The interdependent down-regulation of EGFR, CD44 and EMMPRIN in the three breast carcinoma cell lines attenuated the invasion tendency, as demonstrated by the scratch test and zymography although this effect was less evident in MDA-MB-231 cells.

IIF, individually given, was found to have off-target effects on EGFR, CD44 and EMMPRIN. These new molecular targets enhanced IIF’s potential as an antineoplastic drug. Apart from its ability to bind RXR and selectively activate RXRγ, IIF was demonstrated to have a greater antiproliferative effect than ATRA and 9-*cis-*retinoic acid in the leukemic cell line HL-60. IIF also induced differentiation in several cancer cell lines, inhibited MMP-2 and MMP-9 and increased expression of their inhibitors (TIMP-1 and TIMP-2) [44]. Both ATRA and IIF down-regulated genes that supported the tumour stem cell phenotype maintenance (Slug, Notch-3 and Jagged-1) by blocking the NF-κB axis: these events were associated with re-expression of differentiation markers such as ERα and keratin-18 in breast carcinoma stem cells [45].

## Conclusion

**Figure 7:**
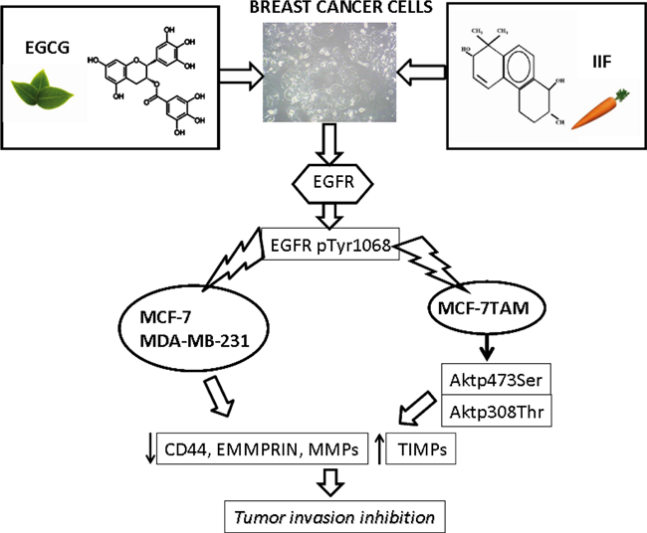
Mechanism of action of EGCG and IIF in MCF-7, MCF-7TAM and MDA-MB-231 cells p1068EGFR down-regulation occurred in all the investigated cell lines and it was associated with AKT inactivation in MCF-7TAM. Cancer cell markers of invasion were down-regulated as well.

We demonstrated that EGCG and IIF in combination exert a strong anti-invasion effect on breast carcinoma cells and can limit the expression of molecules related to the development of an invasive phenotype in breast carcinoma cells with distinct biomolecular characteristics, including a cell line resistant to tamoxifen and a cell line representing the triple-negative breast cancer phenotype. These events occurred in association with AKT inactivation in MCF-7TAM cells and independent of AKT activity in MCF-7 and MDA-MB-231 cells (Figure 7). Furthermore, IIF, individually given, was found to down-regulate EGFR p1068 phosphorylation, CD44 and EMMPRIN expression. The association of a multitarget molecule like EGCG and a molecule with interesting off-target effects like the rexinoid IIF also has the advantage of being less toxic than most antineoplastic drugs currently employed in cancer therapy. Phytochemicals, such as EGCG, can be effective towards numerous molecules involved in cancer onset and development, limiting the drug resistance so often responsible for relapse and therapeutic failure. The association of phytochemicals with a synthetic molecule like IIF, characterized by relative toxicity and interesting antineoplastic properties, can meet the need for therapeutic strategies with multifocal targets. Combining molecules able to interact with different molecular pathways and with limited side-effects represent a promising strategy that ought to be pursued because of the significant individual, economic and social affects of cancer.

## Abbreviations

ATRA: all *trans*-retinoic acid
CSC: cancer stem cell
DABCO: diazabicyclo[2.2.2]octane
EGCG: epigallocatechin-3-gallate
EGFR: epidermal growth factor receptor
EMMPRIN: extracellular matrix metalloproteinase inducer
EMT: epithelial–mesenchymal transition
ERα: oestrogen receptor α
HA: hyaluronic acid
IIF: 6-OH-11-O-hydroxyphenanthrene
MMP: matrix metalloproteinase
PI3K: phosphatidylinositol-3-kinase
RT-PCR: reverse transcription-PCR
RXRγ: retinoid X receptor-γ
TIMP: tissue inhibitor of metalloproteinase
